# Seroprevalence of IgG Antibodies Against *Toxoplasma gondii* in HIV-Infected and Non-Infected Population in Iquitos, Peru

**DOI:** 10.3390/pathogens15040374

**Published:** 2026-04-01

**Authors:** Margot Faustino, Carlos Alonso Flores, Edith S. Málaga-Machaca, Luis Canales, Juan Jiménez-Chunga, Patricia Sheen, Maritza Calderón

**Affiliations:** 1 Laboratorio de Investigación en Enfermedades Infecciosas, Universidad Peruana Cayetano Heredia (UPCH), Lima 15102, Peru; delia.faustino.a@upch.pe (M.F.); carlos.flores.b@upch.pe (C.A.F.); edithmalaga@gmail.com (E.S.M.-M.); 2 Laboratorio de Bioinformática y Biología Molecular, Facultad de Ciencias y Ingeniería, Universidad Peruana Cayetano Heredia (UPCH), Lima 15102, Peru; luis.canales@upch.pe (L.C.); patricia.sheen@upch.pe (P.S.); 3 Laboratorio de Parasitología Molecular y Celular, Facultad de Ciencias Bilógicas, Universidad Nacional Mayor de San Marcos (UNMSM), Lima 15081, Peru; jjimenezc@unmsm.edu.pe

**Keywords:** *Toxoplasma gondii*, seroprevalence, HIV

## Abstract

Toxoplasmosis is a cosmopolitan zoonosis that particularly threatens pregnant women, their fetuses and immunocompromised individuals. Among people living with HIV, *Toxoplasma gondii* may invade the central nervous system, producing neuropathological effects associated with mental and psychiatric disorders. We assessed the seroprevalence of anti-*T. gondii* IgG in HIV-infected and HIV-uninfected residents of Iquitos, Peru, and evaluated an in-house ELISA based on total lysate antigen (TLA) and recombinant GRA1 (rGRA1), with ELISA-TLA compared against a commercial kit. In this observational cross-sectional study, 151 participants were enrolled: 92 HIV-positive and 59 HIV-negative. ELISA-TLA showed a seroprevalence of 88.08% (133/151), reaching 91.30% (84/92) in the HIV-positive group and 83.05% (49/59) in the HIV-negative group. ELISA-rGRA1 showed a similar epidemiological pattern but lower overall seroprevalence, 81.46% (123/151), with 84.78% (78/92) in HIV-positive and 74.58% (44/59) in HIV-negative participants. Taken together, both TLA and rGRA1-based ELISAs showed similar epidemiological patterns, supporting the consistency of the serological findings. These results also indicate very high exposure to *T. gondii* in Iquitos, particularly among HIV-positive individuals, in whom prior exposure is clinically relevant because of the risk of reactivation under immunosuppression. Serological screening and preventive counseling may therefore be warranted in high-burden Amazonian communities.

## 1. Introduction

Toxoplasmosis is a cosmopolitan zoonosis that affects most warm-blooded animals, including humans. It comprises an acute phase and a chronic phase. People acquire the infection by consuming raw or undercooked meat, water, vegetables, or fruits contaminated with oocysts, through accidental inoculation, transfusion/transplantation, or congenitally [1,2]. The etiological agent is *Toxoplasma gondii* (*T. gondii*), an intracellular protozoan that has two adaptable stages in humans for its survival and propagation [3,4,5,6]. Tachyzoites are the rapidly multiplying form that disseminates, and bradyzoites are the form that encysts in human tissues [2,7].

Toxoplasmosis is an infection that almost always goes unnoticed in immunocompetent people, but it poses a risk to pregnant women, their fetuses and immunocompromised individuals due to the risk of primary infection or reactivation [8]. In individuals infected with the Human Immunodeficiency Virus (HIV), tachyzoites can spread through the bloodstream to various organs, such as the brain, eyes, and muscles, causing fatal complications [9,10].

The most common and most severe manifestation of the disease is toxoplasmic encephalitis, and latent toxoplasmosis is also responsible for neuropathological effects that cause mental and psychiatric disorders due to the active proliferation of the parasite within the brain, such as anxiety, schizophrenia spectrum disorders, depression, and suicidal behavior [11]. In patients with HIV, the risk of toxoplasmosis reactivation depends on access to antiretroviral therapy and the patient’s immune status. Reactivation can occur when the CD4 count is generally less than 200 cells/mm^3^ and it occurs in up to 30% of patients, causing serious complications [11,12].

Detection of *T. gondii* is routinely performed by serological methods, which use parasite antigens such as surface antigens (SAG), dense granule antigens (GRA), rhoptry antigens (ROP), and microneme antigens (MIC); these may be either native or recombinant. A native antigen, such as Total Lysate Antigen (TLA), can be obtained through culture, and subsequent complete disruption of the tachyzoites releases the parasite proteins and other antigenic components [13]. An antigen such as GRA1, which consists of proteins secreted by the parasite after invading the cell, can be obtained in recombinant form through genetic engineering using an expression system that is generally bacterial [10]. Evaluations of a test for epidemiological studies are based on the performance parameters of a diagnostic test; they determine the test’s ability to identify the presence or absence of a disease, using indicators such as sensitivity, specificity, predictive values, and diagnostic accuracy, assessed in relation to a reference test [14,15,16].

Approximately 30% of the world’s population is infected with *T. gondii* [17]. In South America, the seroprevalence of toxoplasmosis can range from 7% to 38% [17,18]. These variations can mainly be explained by geographic region, dietary habits, sanitation, and host susceptibility [16]. Surveillance for toxoplasmosis is limited in Peru. The latest national report, from 2022, shows a seroprevalence of 45.2% (498/1101) [19]. However, it is not possible to identify or differentiate the percentage of at-risk populations, such as people with HIV.

Over the last 3 years (2023–2025), studies have focused on the Peruvian Amazon as the main hotspot for cases with high seroprevalence, especially in people with HIV, and have reported a seroprevalence between 71% and 97% [20,21,22,23,24]. The elevated seroprevalence values reflect the importance of conducting surveillance in at-risk populations, especially in the Peruvian Amazon. In this regard, the main objective of this study was to determine the seroprevalence of IgG against *T. gondii* in an HIV-positive group and an HIV-negative group in the city of Iquitos, Peru.

## 2. Materials and Methods

### 2.1. Ethical Statement

This study was approved by the Ethics Committee of the Peruvian University Cayetano Heredia, registered with code numbers 62495 and 201148. Samples were collected only after obtaining informed consent from the participants or a health care proxy.

### 2.2. Study Design and Study Sites

An observational, cross-sectional study was conducted to detect IgG antibodies against *T. gondii* in sera from HIV-infected and non-infected individuals using an in-house ELISA test with total lysate antigen (TLA) from the *T. gondii* RH strain. Participants were recruited in Iquitos, the capital of the Loreto department in the Peruvian Amazon, located in the northeast of Peru. Geographically, it is a riverine plain with a tropical and humid climate, experiencing constant rainfall throughout the year and temperatures ranging from 21 °C to 33 °C [25].

### 2.3. Participants Recruitment and Collection of Information

Participants were recruited from two different settings in Iquitos, Peru. Eligible participants in both groups were adults aged ≥18 years who provided informed consent, or whose legal representative provided consent when applicable. HIV-positive individuals were enrolled at Hospital Regional de Loreto between 28 October 2020 and 24 February 2023 as part of an ongoing study conducted among people living with HIV, including both hospitalized and outpatient individuals. Some participants presented neurological syndromes, whereas others did not; this was considered a clinical characteristic and not an inclusion criterion. HIV-negative participants were enrolled in 2023 from the general population through home visits as part of a community-based household study on tick-borne disease in households with dogs. In that study, 286 households in Iquitos were randomly selected and visited. Eligible HIV-negative participants were residents of Iquitos, lived in households with dogs, and had no symptoms of illness at the time of enrolment.

We collected a total of 151 serum samples. Of these, 92 were from HIV-positive individuals and 59 from HIV-negative individuals. Samples were categorized by sex and into three age groups: young adults (18–35 years), adults (36–60 years), and older adults (61–78 years).

### 2.4. Laboratory Tests for HIV

Hospitalized patients were tested for HIV by the “Programa de Control de Enfermedades de Transmisión Sexual y SIDA (PROCETSS)”. According to this program, a commercially available ELISA (AccuBioTech, Beijing, China) was used as a screening test, and Western blot was employed as a confirmatory test. Non-hospitalized individuals were tested for HIV using the INSTI HIV Self-Test (bioLytical Laboratories Inc., Richmond, BC, Canada), which has a sensitivity of 100% and a specificity of 99.5%.

### 2.5. rGRA1 Expression and Purification

The coding region of *Toxoplasma gondii* GRA1 (RH strain; ToxoDB locus TGRH88_081130) was amplified from parasite cDNA using primers containing NcoI and XhoI restriction sites (Fw, 5’-TAACCATGGGAGTGCGTGTGAGCGCTATTG-3’; Rv, 5’-TTACTCGAGCTCTCTCTCCTGTTAGGAACCCA-3’) and cloned into pET-28a to generate an N-terminal His-tagged construct. The recombinant plasmid was transformed into Escherichia coli BL21(DE3) pLysS, and protein expression was induced in LB medium supplemented with kanamycin and chloramphenicol at OD600 = 0.6 using 0.5 mM IPTG for 4 h at 37 °C. Cells were lysate, and the soluble fraction was purified by nickel-affinity chromatography (HisTrap HP column), with rGRA1 eluting predominantly at 300 mM imidazole. The purified protein was concentrated and buffer-exchanged into PBS containing 20% glycerol. Expression and identity were confirmed by SDS-PAGE and anti-His Western blot. The final recovered protein concentration was 0.6 µg/µL, corresponding to a total yield of 270 µg (approximately 0.54 mg/L of bacterial culture).

### 2.6. In-House ELISA TLA and rGRA1 Test

Seroprevalence was determined using an in-house ELISA. Total lysate antigens (TLA) of *T. gondii* were obtained via cell culture following the protocols of Flores et al., 2025 [20]. Tachyzoites of the RH strain of *T. gondii* were cultured in LLC-MK2 cell lines. The ELISA using TLA (ELISA-TLA) for IgG detection against *T. gondii* was validated against a commercial ELISA (Vircell Microbiologist (Granada, Spain), code G1027), which uses native antigens from the RH strain of *T. gondii*. Positive and negative concordance rates were 98.3% and 100%, respectively.

Polystyrene plates (Nunc Nalgene, Rochester, NY, USA) were sensitized with TLA at a concentration of 1 µg/mL in 0.05 M bicarbonate-carbonate buffer (C3041; Sigma-Aldrich, St. Louis, MO, USA). Free binding sites were blocked with skim milk in Phosphate-Buffered Saline (PBS) 1X + 0.05% Tween 20. The primary antibody was used at dilutions of 1:500 for TLA and 1:100 for rGRA1. HRP-conjugated anti-human IgG antibody (SeraCare Life Sciences, Milford, MA, USA) was then add-ed as the secondary antibody at dilutions of 1:10,000 for TLA and 1:7500 for rGRA1, and incubated at 37 °C for 1 h. Readings were taken at 450 nm using an ELISA reader. Each sample was run in duplicate, and each plate included a pool of four negative controls, three positive controls, and a blank. The cut-off value was calculated as the mean of the negative controls plus three standard deviations.

For ELISA quality control, serum samples were distributed across plates at random and analyzed in a blinded manner, and each sample was tested in duplicate. High-positive, low-positive, and negative pooled serum controls were included in each plate. Cut-off values were defined globally for each antigen and were not recalculated on a per-plate basis. Both antigens were produced in-house, stored in 1X PBS, and monitored by SDS-PAGE as part of batch quality control.

### 2.7. Data Analysis

The study population (n = 151) was described by summarizing categorical variables (HIV infection status, sex, and age groups) as absolute frequencies and percentages. Optical density (OD450) values were summarized as means, standard deviations, and 95% confidence intervals. Associations between anti-*T. gondii* IgG seropositivity and categorical variables were assessed using Pearson’s chi-square test or Fisher’s exact test, as appropriate. For continuous OD450 data, comparisons according to HIV infection status and sex were performed separately among seropositive and seronegative samples. Comparisons between two independent groups were performed using a two-tailed unpaired Student’s *t* test when parametric assumptions were met, and with the exact two-tailed Mann–Whitney U test otherwise. Comparisons among more than two age groups were performed using the Kruskal–Wallis test with Dunn’s post hoc multiple-comparisons procedure. A two-sided *p* value < 0.05 was considered statistically significant.

## 3. Results

### 3.1. Demographic and Clinical Characteristics of the Study Population

Of the 151 serum samples collected, 60.93% (92/151) were from individuals infected with HIV, while 39.07% (59/151) were from HIV-negative individuals. 58.28% (88/151) of participants were male and 41.72% (63/151) were female, 49.67% (75/151) of participants were between 36 and 60 years of age, 46.36% (70/151) were in the 18–35 years group, and only 3.97% (6/151) of participants were aged 61–78 years (Table 1).

### 3.2. rGRA1 Expression

**Figure 1:**
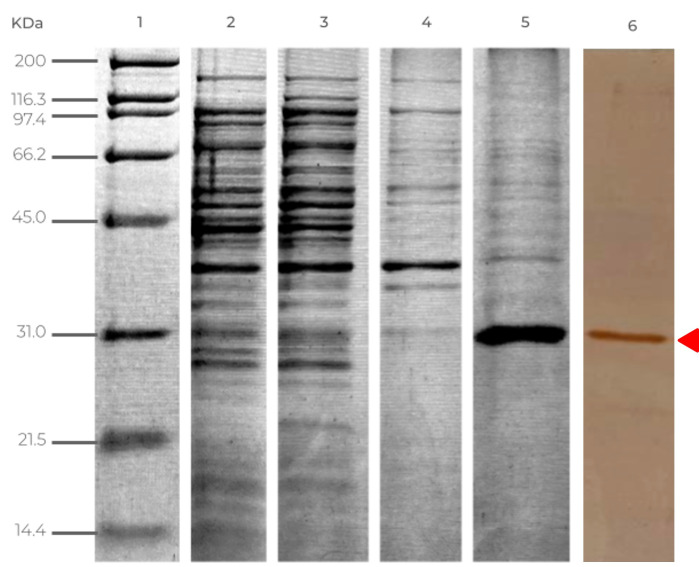
SDS-PAGE and anti-His Western blot analysis of rGRA1 expression and purification in *E. coli* BL21(DE3) pLysS. Lanes: **1**, broad-range molecular weight marker; **2**, non-induced extract; **3**, induced extract; **4**, eluate with 100 mM imidazole; **5**, eluate with 300 mM imidazole; **6**, anti-His Western blot of the 300 mM eluate. The band corresponding to rGRA1 was detected at approximately 31 kDa and was enriched predominantly in the fraction eluted with 300 mM imidazole. The anti-His immunoblot showed a reactive band at approximately 31 kDa (indicated by the red triangle), confirming the identity of the purified recombinant protein.

Heterologous expression of rGRA1 in *E. coli* BL21(DE3) pLysS yielded a predominant species of approximately 31 kDa, which was detectable in the induced lane of the SDS-PAGE gel. During IMAC purification, no appreciable recovery of the protein of interest was observed in the fraction eluted with 100 mM imidazole, whereas elution with 300 mM imidazole produced a clearly enriched fraction dominated by an intense band that co-migrated with the species detected in the induced extract. Collectively, these results demonstrate the inducible expression, nickel-affinity recovery, and effective enrichment of rGRA1 from the soluble fraction. Following concentration and buffer exchange, the purified protein was recovered at a final concentration of 0.6 µg/µL in a total volume of 450 µL, corresponding to 270 µg of total protein and an approximate yield of 0.54 mg/L of bacterial culture (Figure 1).

### 3.3. Seroprevalence of Toxoplasmosis in the Study Population

**Figure 2:**
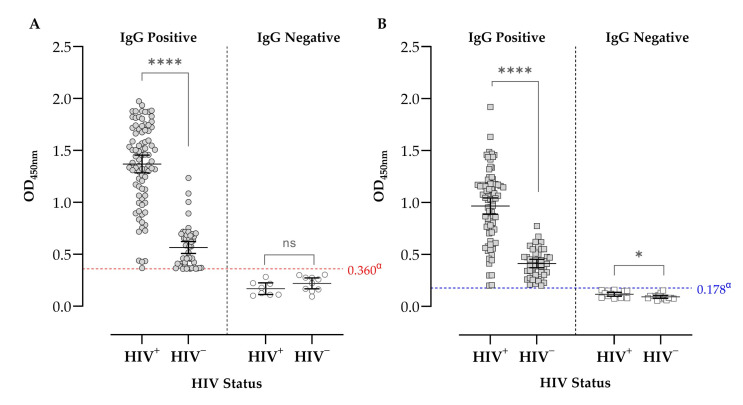
Seroprevalence of IgG antibodies against *T. gondii* according to HIV infection status. Each circle represents the OD450 result obtained using the in-house ELISA test. (**A**) The cut-off indicated by dashed red lines, established at 0.360 (0.156 + (3 × 0.068))^α^ for TLA. Among IgG-positive results, a significant difference was observed between HIV-infected and non-infected groups (*p* < 0.0001). (**B**) The cut-off indicated by dashed blue lines, established at 0.178 (0.104 + (3 × 0.025))^α^ for rGRA1. Among IgG-positive and IgG-negative results, a significant difference was observed between the HIV-infected and non-infected groups in both TLA and rGRA1 (*p* < 0.0001). Regarding IgG-negative results using only rGRA1, a significant difference was observed between the HIV-infected and non-infected groups (*p* = 0.0495). ^α^ The cut-off (red and blue dashed line) was defined as the mean OD450 of negative samples plus 3 standard deviations. The statistical values in the HIV status comparisons are shown as follows: **** indicates *p* < 0.0001, * indicates 0.0332 ≤ *p* ≤ 0.1233, and ns indicates *p* ≥ 0.1234.

Using the ELISA-TLA assay, the overall seroprevalence of anti-*T. gondii* IgG in the study population was 88.08% (133/151). Overall, 60.93% (92/151) of participants were HIV-positive, and IgG seroprevalence was significantly higher in the HIV-positive group (91.30% [84/92]) than in the HIV-negative group (83.05% [49/59]). In HIV-positive participants, mean OD450 values were 1.37 ± 0.393 (95% CI: 1.28–1.45) among seropositive individuals and 0.169 ± 0.0668 (95% CI: 0.113–0.224) among seronegative individuals, whereas in HIV-negative participants the corresponding mean OD450 values were 0.565 ± 0.200 (95% CI: 0.508–0.622) and 0.220 ± 0.0734 (95% CI: 0.167–0.272), respectively (Figure 2A). HIV infection was present in 63.16% (84/133) of IgG-Anti-*T. gondii* seropositive participants and in 44.44% (8/18) of IgG- Anti-*T. gondii* seronegative participants. On the other hand, using the ELISA-rGRA1 assay, the overall seroprevalence of anti-*T. gondii* IgG in the study population was 81.46% (123/151). Overall, 60.93% (92/151) of participants were HIV-positive, and IgG seroprevalence was higher in the HIV-positive group (84.78% [78/92]) than in the HIV-negative group (74.58% [44/59]), although this difference was not statistically significant (*p* = 0.180). HIV infection was present in 63.93% (78/122) of IgG anti-*T. gondii* seropositive participants and in 48.28% (14/29) of seronegative participants (Figure 2B).

**Figure 3:**
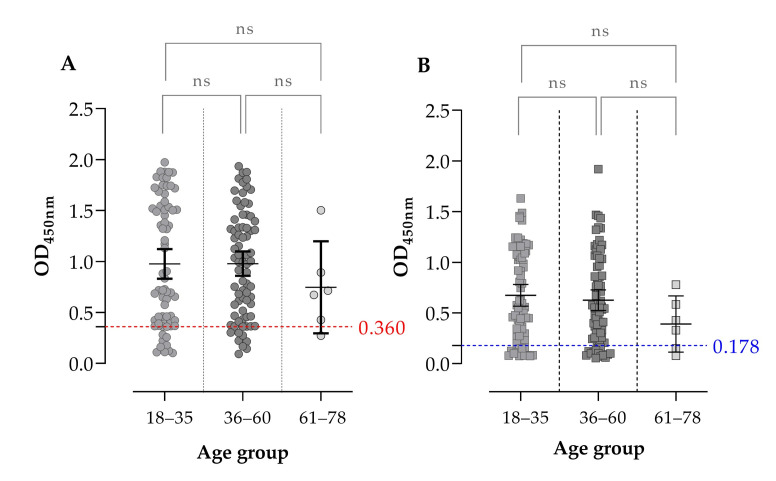
Distribution of IgG antibodies against *T. gondii* according to age in the general population. (**A**) ELISA-TLA and (**B**) ELISA-rGRA1 according to age group (18–35 (n: 70), 36–60 (n: 75), and 61–78 (n: 6) years). No significant differences in OD450 distributions were observed among age categories for either assay (Kruskal–Wallis test). Dashed lines indicate the assay cut-off values (0.178 for ELISA-rGRA1 and 0.360 for ELISA-TLA). Each point represents one serum sample.

Analysis by age showed that ELISA-TLA yielded anti-*T. gondii* IgG seroprevalence rates of 87.14% (61/70) in participants aged 18–35 years, 89.33% (67/75) in those aged 36–60 years, and 83.33% (5/6) in the 61–78-year group. ELISA-rGRA1 showed a comparable age-related pattern, with seroprevalence rates of 81.43% (57/70), 81.33% (61/75), and 66.67% (4/6) in the same age groups, respectively. For ELISA-TLA, median OD450 values were 0.782 (IQR: 0.408–1.540) in the 18–35-year group, 0.996 (IQR: 0.468–1.400) in the 36–60-year group, and 0.694 (IQR: 0.389–1.050) in the 61–78-year group. For ELISA-rGRA1, the corresponding median OD450 values were 0.567 (IQR: 0.270–1.090), 0.549 (IQR: 0.256–0.969), and 0.378 (IQR: 0.133–0.633), respectively. Despite these numerical differences, OD450 distributions did not differ significantly across age categories for either assay, as determined by Kruskal–Wallis testing (ELISA-TLA, *p* = 0.6783; ELISA-rGRA1, *p* = 0.3286). Likewise, post hoc pairwise comparisons showed no significant differences between age groups in either assay (Figure 3).

**Figure 4:**
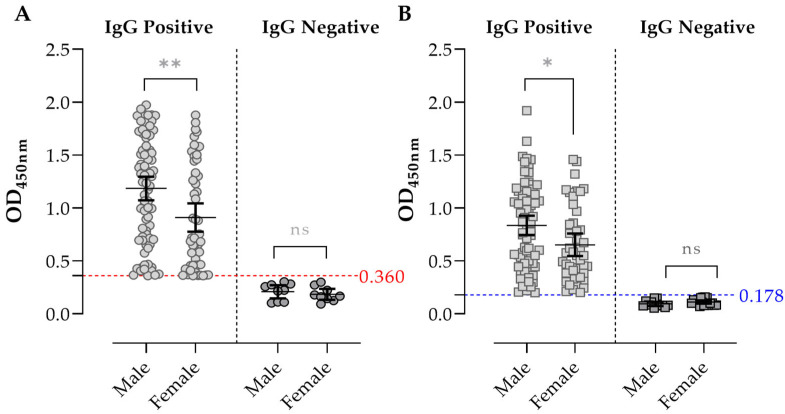
Sex-based distribution of anti-*T. gondii* IgG OD450 values measured by ELISA-TLA and ELISA-rGRA1. (**A**) ELISA-TLA: seroprevalence was 89.77% (79/88) in men and 85.74% (54/63) in women. Among seropositive individuals, median OD450 values were significantly higher in men than in women (1.297 vs. 0.717; *p* = 0.0018), whereas no significant difference was observed among seronegative individuals (0.222 vs. 0.167; *p* = 0.5609). The red dashed line indicates the assay cut-off (OD450 = 0.360). (**B**) ELISA-rGRA1: seroprevalence was 86.36% (76/88) in men and 73.02% (46/63) in women. Among seropositive individuals, median OD450 values were significantly higher in men than in women (0.8635 vs. 0.5495; *p* = 0.0143), while no significant difference was observed among seronegative individuals (0.0825 vs. 0.1020; *p* = 0.1040). The blue dashed line indicates the assay cut-off (OD450 = 0.178). Each point represents one serum sample. The statistical values in the sex comparisons are shown as follows: ** indicates 0.0021 ≥ *p* > 0.0001, * indicates 0.0332 ≤ *p* ≤ 0.1233, and ns indicates *p* ≥ 0.1234.

Regarding sex distribution, ELISA-TLA identified anti-*T. gondii* IgG seroprevalence rates of 89.77% (79/88) in men and 85.74% (54/63) in women. ELISA-rGRA1 showed lower seroprevalence values overall, but the same directional pattern, with 86.36% (76/88) seropositivity in men and 73.02% (46/63) in women. In both assays, seropositive men exhibited higher OD450 reactivity than seropositive women. This difference was significant for ELISA-TLA (*p* = 0.0018) and ELISA-rGRA1 (*p* = 0.0143) by exact two-tailed Mann–Whitney U test. In contrast, no significant sex-related differences were observed among seronegative individuals for either ELISA-TLA (*p* = 0.5609) or ELISA-Rgra1 (*p* = 0.1040) (Figure 4).

## 4. Discussion

The seroprevalence of IgG antibodies against *T. gondii* in the present study was 88.08% (133/151) with ELISA-TLA and 81.46% (123/151) with ELISA-rGRA1, thus supporting a substantial burden of prior exposure in this population across both serological approaches. which is higher than the global average reported in the meta-analysis by Molan et al. (2019), which found a seroprevalence of 25.7% across various populations, including children, adults, pregnant women and their fetuses [17]. Similarly, the study by Sengupta and cols., covering data from 1959 to 2020, reported a global seroprevalence of 36% [26]. Both studies indicate higher prevalence rates in Africa and South America. The high seroprevalence in some regions of South America is associated with living in rural, tropical or subtropical areas, poor sanitary conditions or a deficient immune system [19,27,28].

Iquitos has a tropical climate and is characterized by a lower-middle socioeconomic level, with 8.63% of the population living in poverty [29]. Its geographical isolation from Lima, the capital, exacerbates challenges related to governance, economic development, and access to healthcare [29]. Furthermore, environmental changes due to global warming have affected parasite ecology, with *T. gondii* demonstrating increased oocyst sporulation under environmental stressors [30]. Seasonal rainfall and increased river flow have been shown to facilitate the release of oocysts from soil into water, further exacerbating the spread of the parasite [31]. The higher *T. gondii* seroprevalence observed in our HIV-positive participants may be better understood within a regional context in which HIV transmission and continuity of care remain challenging, rather than as a direct biological effect of HIV on acquisition. Loreto contributes a substantial share of reported HIV cases in Peru, and a multicentre analysis that included Hospital Regional de Loreto showed that only 64.8% of newly diagnosed people living with HIV attended care within 1 month, while 51.6% achieved viral suppression during follow-up, underscoring persistent gaps in timely linkage to care and treatment outcomes [32]. Earlier studies from Amazonian communities in Loreto also documented poor HIV-related knowledge, low acceptance of prevention measures, and self-reported sex between men in 39.7% of participants, together with the absence of condom use in one community, all of which support the presence of structural and behavioral conditions that may sustain HIV transmission in this region [33,34]. In this context, the higher *T. gondii* seroprevalence in the HIV-positive group likely reflects overlapping social vulnerabilities and cumulative exposure in the Peruvian Amazon.

Among the participants included in this study, a higher seroprevalence of *T. gondii* was observed in HIV-infected individuals, at 91.30% (84/92), compared with 83.05% (49/59) among HIV-negative individuals. These findings align with global studies indicating a higher prevalence of latent toxoplasmosis among people with HIV [35,36,37,38]. A similar directional pattern was also observed with ELISA-rGRA1, which showed seroprevalence values of 84.78% (78/92) in HIV-positive individuals and 74.58% (44/59) in HIV-negative individuals, although this difference did not reach statistical significance. The fact that both assays identified higher seroreactivity in the HIV-positive group supports the robustness of the epidemiological pattern observed in this population, even if the recombinant assay yielded lower overall reactivity than ELISA-TLA.

The higher optical density values observed in HIV-positive individuals indicate greater serological reactivity of anti-*T. gondii* IgG antibodies compared with HIV-negative individuals. This pattern is consistent with previous reports describing a high seroprevalence of latent toxoplasmosis in people living with HIV, particularly in settings with limited access to early diagnosis and treatment [20,21,22,23,24]. Accordingly, OD450 values are more appropriately interpreted as markers of serological reactivity and should be considered with caution when inferring antibody concentration, recency of infection, or clinical risk, especially in immunocompromised individuals.

In immunocompromised hosts, the reduced capacity to control parasite replication facilitates persistence and reactivation of *T. gondii*, which exhibits a marked neurotropism and can cause severe organ damage, especially in the central nervous system [9,12,13]. In this context, the high seroprevalence observed among HIV-positive individuals reinforces the potential value of serological screening for identifying prior exposure in populations at risk of reactivation, although serological reactivity alone does not define clinical risk for toxoplasmic encephalitis. That risk is more closely related to factors such as CD4^+^ T-cell count, antiretroviral therapy status, and prophylaxis, which were not evaluated in the present study.

Although OD450 values for ELISA-rGRA1 were lower in absolute terms than those observed with ELISA-TLA, the recombinant assay preserved the same overall trend toward greater serological reactivity in the HIV-positive subgroup, which is consistent with the higher burden of latent infection in immunocompromised hosts.

This issue is particularly relevant for individuals with CD4^+^ T-cell counts < 200 cells/mm^3^. In Latin America, although HIV-related mortality has declined in recent years, the incidence of new infections has increased by approximately 13%. Moreover, an estimated 14% of people living with HIV remain unaware of their infection, and nearly one-third are diagnosed at advanced stages [32]. In regions such as Loreto, one of the Peruvian departments with the highest burden of HIV infection in recent years and a high prevalence of toxoplasmosis, these conditions further increase the risk of opportunistic infections. Epidemiological monitoring of this population using serological screening methods, such as ELISA, is therefore essential, as parasite reactivation in susceptible individuals can lead to severe organ involvement, particularly affecting the central nervous system, with potentially irreversible consequences [11,13]. In this context, our findings suggest that recombinant antigens such as GRA1 may be useful as complementary tools in seroepidemiological screening, especially because recombinant platforms provide improved antigen definition, standardization, and reproducibility; however, when used as single targets, they may still capture a narrower antibody repertoire than native lysate preparations.

Although no statistically significant differences were observed across age groups, higher seroprevalence among adults is consistent with previous studies showing an age-related increase in *T. gondii* IgG positivity, which is generally interpreted as a marker of cumulative exposure rather than age-specific susceptibility [27,37]. This interpretation was supported by both assays in our study, ELISA-TLA showed the highest seroprevalence in the 36–60-year group (89.33%), while ELISA-rGRA1 showed a very similar distribution between the 18–35-year (81.43%) and 36–60-year (81.33%) groups. Although the oldest group showed lower reactivity (66.67%), this finding should be interpreted cautiously given the small number of participants in that subgroup. Likewise, neither assay showed significant differences in OD450 distributions across age categories, suggesting that age in this population is more likely to reflect cumulative environmental exposure than marked differences in humoral response intensity.

Sex-related differences in *T. gondii* seroprevalence remain inconsistent across studies, with some reports indicating higher prevalence in men, while others describe higher rates in women, particularly in the context of pregnancy due to increased screening and heightened clinical awareness [39,40,41]. In the present study, seroprevalence was higher among male participants compared with females. This male predominance was observed with both assays, with ELISA-TLA showing seroprevalence values of 89.77% in men and 85.74% in women, and ELISA-rGRA1 showing a wider difference, with 86.36% in men and 73.02% in women. Higher optical density values observed in males may reflect differences in exposure intensity or cumulative risk rather than sex-specific susceptibility, potentially influenced by behavioral or occupational factors. Importantly, the higher OD450 values in seropositive men were reproduced by both ELISA-TLA and ELISA-rGRA1, whereas no significant sex-related differences were observed among seronegative individuals with either assay. Overall, sex alone is unlikely to represent an independent risk factor for *T. gondii* infection and should be interpreted within the context of environmental exposure and host-related factors.

This study has certain limitations that should be acknowledged. IgG antibodies against *T. gondii* were detected using an in-house indirect ELISA based on TLA, which, although previously validated, may present cross-reactivity with other apicomplexan parasites circulating in endemic regions such as the Peruvian Amazon [42]. In addition, the use of TLA does not allow for discrimination between different stages of infection. Our findings with ELISA-rGRA1 also suggest that, although recombinant antigens can improve assay definition and reproducibility, the use of a single recombinant target may yield lower sensitivity than native lysate preparations, likely because it captures a narrower antigenic repertoire. Future studies incorporating more specific antigens, such as recombinant stage-specific proteins, may improve diagnostic specificity and enable a more refined assessment of infection dynamics [43,44]. Accordingly, recombinant antigens such as GRA1 may be more informative as components of multi-antigen or stage-oriented diagnostic platforms than as standalone replacements for native lysate in high-exposure settings. An additional limitation is that the HIV-positive and HIV-negative groups were recruited from different settings and during partially overlapping periods, which may introduce selection differences and limit the direct comparability of seroprevalence and OD450 values between groups. Furthermore, CD4^+^ T-cell count, antiretroviral therapy status, and prophylaxis data were not available because they were not evaluated in the present study, and we acknowledge this as a limitation when interpreting the clinical implications of the serological findings. Despite these limitations, the high seroprevalence observed highlights the substantial burden of *T. gondii* infection in this setting and supports the relevance of the findings.

## 5. Conclusions

This study demonstrates a high seroprevalence of IgG antibodies against *Toxoplasma gondii* in both HIV-infected and HIV-uninfected individuals in Iquitos, Peru, with a greater burden observed among people living with HIV. These findings highlight the endemic nature of toxoplasmosis in the Peruvian Amazon and underscore the vulnerability of immunocompromised populations in this setting. The similar epidemiological trends identified by both ELISA-TLA and ELISA-rGRA1 strengthen the consistency of these observations, while also indicating that recombinant antigens may complement native lysate-based assays in seroepidemiological studies. Overall, our results support the need for strengthened surveillance and targeted screening strategies in high-risk populations, particularly in regions where environmental exposure and delayed diagnosis may converge to increase the risk of severe disease.

## Figures and Tables

**Table 1:** Characteristics of the Study Population (n = 151).

**Characteristics**	**Total, n (%)**
Age Group	
18–35 years	70 (46.36)
36–60 years	75 (49.67)
61–78 years	6 (3.97)
Sex	
Male	88 (58.28)
Female	63 (41.72)
HIV Infection Status	
Positive	92 (60.93)
Negative	59 (39.07)
Seroprevalence of *T. gondii*	
Using TLA	
Positive	133 (88.08)
Negative	18 (11.92)
Using rGRA1 antigen	
Positive	123 (81.46)
Negative	28 (18.54)

## Data Availability

Data will be made available on request.

## Abbreviations

The following abbreviations are used in this manuscript:

HIV	Human Immunodeficiency Virus
ELISA	Enzyme-Linked Immunosorbent Assay
IgG	Immunoglobulin G
TLA	Total Lysate Antigen
rGRA1	Recombinant Dense Granule Antigen 1
CI	Confidence Interval
OD450	Optical Density at 450 nm
CD4	Cluster of Differentiation 4
